# The impact of preoperative systemic inflammation on the efficacy of intravenous iron infusion to correct anaemia prior to surgery for colorectal cancer

**DOI:** 10.1186/s13741-020-00146-4

**Published:** 2020-06-11

**Authors:** Stephen T. McSorley, John H. Anderson, Thomas Whittle, Campbell S. Roxburgh, Paul G. Horgan, Donald C. McMillan, Colin W. Steele

**Affiliations:** School of Medicine and Dentistry, University Department of Surgery, Academic Unit of Surgery, Glasgow Royal Infirmary, University of Glasgow, Level 2, New Lister Building, Alexandra Parade, Glasgow, G4 0SF UK

**Keywords:** Anaemia, Iron, Colorectal cancer, Inflammation

## Abstract

**Aim:**

Intravenous iron is increasingly used prior to surgery for colorectal cancer (CRC) to correct iron deficiency anaemia and reduce blood transfusion. Its utility in functional iron deficiency (FID) or anaemia of inflammation is less clear. This observational study examined post-iron infusion changes in haemoglobin (Hb) based on grouping by C-reactive protein (CRP) and ferritin.

**Methods:**

Anaemic (M:Hb < 130 mg/L, F:Hb < 120 mg/L) patients with CRC receiving iron infusion, within a preoperative anaemia detection and correction protocol, at a single centre between 2016 and 2019 were included. Patients were grouped by iron deficiency (ferritin < 30 μg/L and CRP ≤ 5 mg/L, *n* = 18), FID (ferritin < 30 μg/L and CRP > 5 mg/L, *n* = 17), anaemia of inflammation (ferritin ≥ 30 μg/L and CRP > 5 mg/L, *n* = 6), and anaemia of other causes (ferritin ≥ 30 μg/L and CRP ≤ 5 mg/L, *n* = 6). Median change in Hb and postoperative day (POD) 1 Hb was compared by Kruskal-Wallis test.

**Results:**

Iron-deficient patients had the greatest increase in Hb after infusion (24 mg/L), highest POD 1 Hb (108 mg/L), and required no blood transfusions. Patients with FID had the second greatest increase in Hb (15 mg/L) and second highest POD 1 Hb (103 mg/L). Those with anaemia of inflammation had little increase in Hb after infusion (3 mg/L) and lower POD 1 Hb (102 mg/L) than either iron-deficient group. Those without iron deficiency showed a decrease in haemoglobin after infusion (− 5 mg/L) and lowest POD 1 Hb (95 mg/L).

**Conclusions:**

Preoperative intravenous iron is less efficacious in patients with anaemia of inflammation and FID undergoing surgery for CRC, compared with true iron deficiency. Further understanding of the role of perioperative iron infusions is required for maximum gain from therapy.

## Introduction

Anaemia and perioperative allogeneic blood transfusion are associated with poor outcomes following colorectal cancer (CRC) surgery (Amato and Pescatori 2006). Perioperative optimisation of haemoglobin levels using iron supplementation in iron-deficient patients has been shown to reduce perioperative blood transfusion requirement in colorectal surgery (Froessler et al. 2016). Furthermore, parenteral iron infusions have been shown to improve preoperative haemoglobin levels to a greater degree than oral iron supplements (Keeler et al. 2017). As a result of these observations, our specialist colorectal surgery unit adopted preoperative iron deficiency anaemia identification and iron infusion protocols in 2015 to optimise patient haemoglobin prior to surgery (Quinn et al. 2017).

Although this form of optimisation targets those patients with hypochromic microcytic anaemia of iron deficiency classically associated with gastrointestinal cancers, there is increasing evidence that normocytic anaemia is twice as prevalent (around 20% at diagnosis, compared to 10% with microcytic anaemia) amongst patients diagnosed with colorectal cancer (McSorley et al. 2019a). Patients with normocytic anaemia had higher levels of inflammation as measured by modified Glasgow Prognostic Score (mGPS) (McSorley et al. 2019b). Furthermore, such normocytic anaemia was associated with poorer outcomes following treatment with curative intent (Vayrynen et al. 2018).

It has been proposed that inflammation disturbs hepcidin-mediated red cell iron binding and promotes a ‘functional’ iron deficiency (FID), where despite sufficient stores, iron cannot be utilised for erythropoiesis, leading to anaemia (Kelly et al. 2017). The same mechanisms cause a reduction in enteral iron absorption and sequestration of iron in the reticuloendothelial system, leading to the possibility that any additional iron supplementation given to these patients will not lead to a corresponding change in haemoglobin concentration or red cell count (Nemeth and Ganz 2014).

Therefore, the aim of the present study was to determine if any patients with apparent anaemia of inflammation, or FID, had been treated with preoperative parenteral iron supplementation in our unit and, if so, to determine the impact on haematological parameters. We hypothesised that the presence of inflammation would perturb iron uptake by cells and limit preoperative response to iron infusion. The present study utilised a prospectively collected cohort of primary colorectal cancer patients, given preoperative iron infusions as determined by individual consultants in adherence to unit protocol.

## Patients and methods

### Patients

Patients with primarily operable colorectal cancer receiving preoperative iron infusion followed by surgery in a single centre between January 2016 and January 2019 were studied. Anonymised clinicopathological data were prospectively recorded in a secure database and subsequently analysed. Patients undergoing surgery for benign disease, with a diagnosed inflammatory disorder or vasculitis, or an underlying haematological malignancy were not included. Patient demographics, along with TNM stage (TNM, 5th ed, AJCC), tumour site, American Society of Anesthesiology (ASA) grade, and surgical approach, were recorded.

**Fig. 1:**
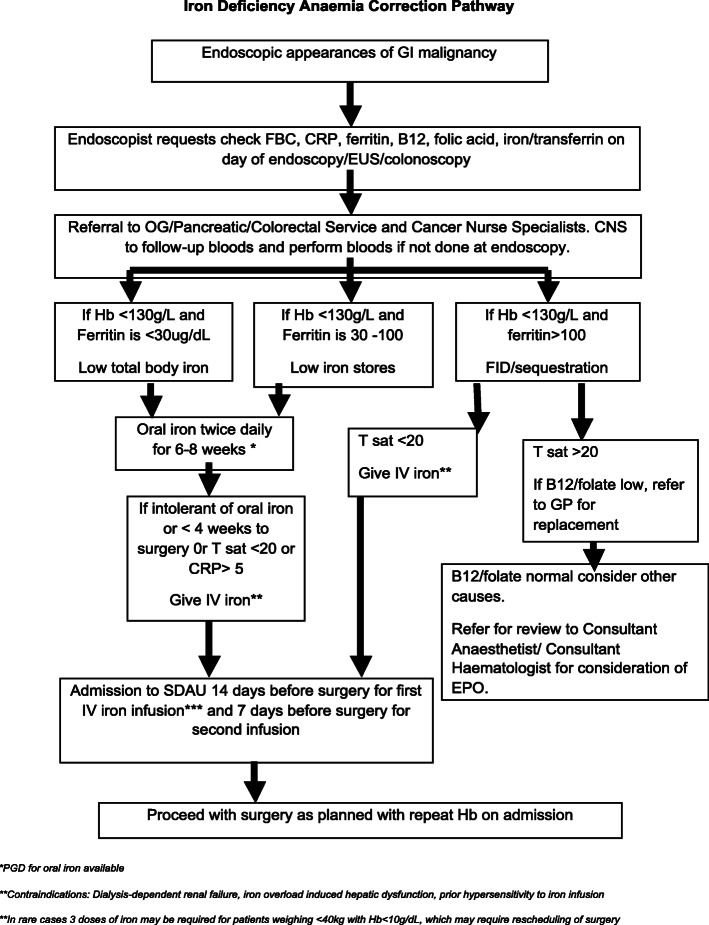
Iron infusion pathway adopted in Glasgow Royal Infirmary. CNS, cancer nurse specialist; CRP, C-reactive protein; EPO, erythropoietin; FBC, full blood count; FID, functional iron deficiency; GP, general practitioner; Hb, haemoglobin; IV, intravenous; T sat, transferrin saturation; SDAU, same day admission unit

All patients were treated by 11 consultant colorectal surgeons in a single institution. All cases were discussed at a specialist colorectal oncology multidisciplinary team meeting, before and after surgery. Each patient was cared for according to unit standardised ERAS protocols and managed by a combination of laparoscopic or open surgery dependent on surgeon preference. Treating surgeons were responsible for identification of anaemia in their patients and determining whether this anaemia required treatment with iron infusion. Treating physicians were encouraged to utilise protocols developed within the unit by surgeons and anaesthetists (Fig. 1).

A waiver of need for ethical approval was granted by the West of Scotland Research Ethics Committee. Caldicott Guardian approval for the collection and analysis of anonymised patient data as part of this project was granted by the office of the Caldicott Guardian for the NHS Greater Glasgow and Clyde region.

### Methods

Haematological parameters—full blood count (FBC)-derived haemoglobin and mean corpuscular volume (MCV), ferritin, transferrin saturation (TSAT)—and the biochemical inflammatory parameter C-reactive protein were measured at the time of endoscopic diagnosis and recorded prior to any subsequent parenteral iron infusion, with the purpose of detecting iron deficiency anaemia. Patients were classified as having anaemia based on WHO guidelines for males, haemoglobin (Hb) < 130 mg/L, and females, Hb < 120 mg/L (WHO 2011). Furthermore, anaemic patients were classified as having iron deficiency based on ferritin < 30 μg/L, ferritin 30–100 μg/L with TSAT < 20% or CRP > 5 mg/L, or ferritin > 100 μg/L and transferrin saturation < 20% as shown above (Fig. 1).

Serum concentrations of CRP (mg/L) were measured by the immunoturbidimetric method in the clinical biochemistry laboratory using an autoanalyser (Architect; Abbot Diagnostics, Maidenhead, UK) with a lower detectable limit of 0.2 mg/L. Ferritin was analysed using a 2-step chemiluminescent microparticle immunoassay in the clinical haematology laboratory. There were no sustained concerns regarding IQC performance requiring investigation into the performance of the assays. The A, B, and C scores were within the EQA (UK NEQAS) targets during the study period.

Patients had FBC samples taken on the day of surgery and again on postoperative day 1. The change in haemoglobin following iron infusion was calculated by subtracting immediate pre-operative haemoglobin from haemoglobin at presentation.

Patients deemed suitable underwent administration of intravenous ferric carboxymaltose (Ferinject®, Vifor Pharma UK), via peripheral intravenous cannula over 15 min, in the preoperative assessment unit. The dose was dependent on body weight and haemoglobin (Vifor Pharma n.d.), and the aim was to have the first infusion at least 14 days before surgery.

### Statistical analysis

Patients were grouped by protocol-defined pre-infusion ferritin and CRP cut-off values into those who were truly iron deficient (ferritin < 30 μg/L and CRP ≤ 5 mg/L, *n* = 18), those with probable FID (ferritin < 30 μg/L and CRP > 5 mg/L, *n* = 17), those with probable anaemia of inflammation (ferritin ≥ 30 μg/L and CRP > 5 mg/L, *n* = 6), and those with anaemia of another cause who were given parenteral iron despite no evidence of iron deficiency (ferritin ≥ 30 μg/L and CRP ≤ 5 mg/L, *n* = 6). Comparisons were made between categorical clinical and pathological variables using the chi-square test and chi-square test for trend where appropriate. Comparisons were made with pre-infusion haemoglobin, change in haemoglobin, and postoperative day 1 haemoglobin using the Kruskal-Wallis test.

Univariate linear regression was used to examine associations between these variables and change in haemoglobin (Δ Hb mg/L) following iron infusion. Those variables found to be associated with a significance level of *p* < 0.01 were entered into a multivariate linear regression backward condition model to examine independent association. Results were reported as the regression coefficient for each variable and their 95% confidence interval (CI).

Two-sided *p* values < 0.05 were considered statistically significant. All analyses were performed using IBM SPSS version 25 (IBM, Chicago, Illinois, USA).

## Results

### Patients

Over the study period, 52 patients received an intravenous iron infusion prior to elective surgery for colorectal cancer. Patient demographics and clinical, pathological, and haematological variables are shown in Table 1. The majority were male (*n* = 30, 58%) and 65 years or older (*n* = 43, 83%). Laparoscopic resection was performed in 27 (52%), and 45 (86%) had colonic cancer while the remaining 7 (14%) had a rectal cancer. All patients were anaemic prior to iron infusion, and of these, 49 had a pre-infusion ferritin measured, 48 had a pre-infusion CRP measured, and 47 had both measured. Thirty-six (74%) had protocol defined low ferritin while half of the 48 patients who had CRP measured prior to iron infusion had protocol defined systemic inflammation present with a CRP > 5 mg/L.

**Table 1:** Demographic and clinicopathological variables of patients receiving intravenous iron infusions prior to elective colorectal surgery for cancer (n = 52)

Characteristic	*n* (%)
Total	52 (100)
Patient characteristics	
Sex	
Male	30 (58)
Female	22 (42)
Age (years)	
< 65	9 (17)
65–75	15 (29)
> 75	28 (54)
ASA	
1	2 (4)
2	26 (50)
3	22 (42)
4	2 (4)
Pathologic and surgical characteristics	
TNM stage	
1	7 (12)
2	24 (47)
3	18 (35)
4	3 (6)
Tumour site	
Right colon	38 (72)
Left colon	7 (14)
Rectum	7 (14)
Approach	
Open	25 (48)
Laparoscopic	27 (52)
Haematological variables	
Pre-infusion Hb (mg/L)	
Median (range)	103 (65–123)
Pre-infusion anaemia*	
Yes	52 (100
No	0 (0)
Pre-infusion ferritin (μg/L)	
Median (range)	18 (3–302)
< 30	36 (74)
30–100	7 (14)
> 100	6 (12)
Pre-infusion CRP (mg/L)	
Median (range)	6 (1–113)
≤ 5	24 (50)
> 5	24 (50)
Total IV iron dose (mg)	
1000	17 (36)
1500	21 (45)
2000	9 (19)
Days from infusion to surgery	
Median (range)	24 (1–83)
Haematological outcomes	
Post-infusion change (∆) in Hb (mg/L)	
Median (range)	12 (− 15 to 63)
Anaemia ‘corrected’^$^	
Yes	8 (15)
No	44 (85)

*ASA* American Society of Anesthesiology, *CRP* C-reactive protein, *IV* intravenous

*Pre-iron infusion haemoglobin: males < 130 mg/L, females < 120 mg/L

^$^Post-iron infusion haemoglobin: males ≥ 130 mg/L, females ≥ 120 mg/L

### Iron infusion

The total dose of iron infused (Table 1) varied between 1000 mg (*n* = 19, 36%), 1500 mg (*n* = 23, 45%), and 2000 mg (*n* = 11, 19%). The median time between infusion and surgery was 24 days (range 1–83). Following infusion, in the cohort as a whole, the median haemoglobin change before surgery was a rise of 12 mg/L (range − 15 to 63), with haemoglobin rising above the cut-off for anaemia in 8 patients (15%).

### Associations between pre-iron infusion iron status and clinicopathological variables

When patients were grouped by protocol-defined pre-infusion ferritin and CRP cut-off values into those who were truly iron deficient (ferritin < 30 μg/L and CRP ≤ 5 mg/L, *n* = 18), those with probable FID (ferritin < 30 μg/L and CRP > 5 mg/L, *n* = 17), those with probable anaemia of inflammation (ferritin ≥ 30 μg/L and CRP > 5 mg/L, *n* = 6), and those who were given parenteral iron despite no evidence of iron deficiency (ferritin ≥ 30 μg/L and CRP ≤ 5 mg/L, *n* = 6), there was no association between this pre-infusion iron status and sex, age, comorbidity, or disease stage (Table 2).

**Table 2:** Clinicopathological and haematological factors associated with protocol-defined iron status prior to intravenous iron infusion for elective colorectal surgery for cancer (*n* = 47)

Characteristic	Pre-infusion iron status				*p*
	IDA (CRP ≤ 5 + Fer < 30) *n* (%)	FID (CRP > 5 + Fer < 30) *n* (%)	AOI (CRP > 5 + Fer ≥ 30) *n* (%)	Replete/other (CRP ≤ 5 + Fer ≥ 30) *n* (%)	
Total	18 (38)	17 (38)	6 (12)	6 (12)	–
Patient characteristics					
Sex					
Male	13 (72)	10 (59)	1 (17)	3 (50)	0.120
Female	5 (28)	7 (41)	5 (83)	3 (50)	
Age (years)					
< 65	1 (6)	5 (29)	1 (17)	1 (17)	0.261
65–75	6 (33)	2 (12)	3 (50)	3 (50)	
> 75	11 (61)	10 (59)	2 (33)	2 (33)	
ASA					
1	0 (0)	2 (12)	0 (0)	0 (0)	0.070
2	9 (50)	3 (17)	6 (100)	4 (67)	
3	8 (44)	11 (65)	0 (0)	2 (33)	
4	1 (6)	1 (6)	0 (0)	0 (0)	
Pathologic characteristics					
TNM stage					
1	1 (6)	3 (18)	0 (0)	2 (33)	0.448
2	11 (61)	5 (29)	3 (50)	3 (50)	
3	6 (33)	8 (47)	3 (50)	1 (17)	
4	0 (0)	1 (6)	0 (0)	0 (0)	
Tumour site					
Right colon	8 (44)	17 (100)	5 (83)	5 (83)	0.006
Left colon	3 (17)	0 (0)	1 (17)	1 (17)	
Rectum	7 (39)	0 (0)	0 (0)	0 (0)	
Infusion variables					
Total IV iron dose (mg)					
1000	7 (44)	6 (40)	0 (0)	3 (50)	0.254
1500	4 (25)	7 (47)	4 (67)	3 (50)	
2000	5 (31)	2 (13)	2 (33)	0 (0)	
Days from infusion to surgery					
Median (range)	20 (3–83)	30 (7–62)	25 (19–44)	16 (2–48)	0.233
Haematological variables					
Pre-iron Hb (mg/L)					
Median (range)	100 (77–118)	102 (65–122)	110 (75–117)	110 (103–115)	0.101
Δ Hb (mg/L)					
Median (range)	24 (− 6 to 48)	15 (− 15 to 63)	3 (− 11 to 34)	− 5 (− 10 to 8)	0.017
POD1 Hb (mg/L)					
Median (range)	108 (87–133)	103 (87–138)	102 (74–113)	95 (85–99)	0.028

*AOI* anaemia of inflammation, *ASA* American Society of Anesthesiology, *CRP* C-reactive protein (mg/L), *Fer* ferritin (μg/L), *FID* functional iron deficiency, *Hb* haemoglobin (g/L), *IDA* iron deficiency anaemia, *IV* intravenous, *POD* postoperative day

Only tumour site was significantly associated with pre-infusion iron status (*p* = 0.006), with all of the rectal cancer patients in the cohort (*n* = 7, 39% of all iron deficiency patients) having true iron deficiency, while there was a preponderance to right-sided lesions in the functional iron deficiency (*n* = 17, 100%), anaemia of inflammation (*n* = 5, 83%), and non-deficient (*n* = 5, 83%) groups.

### Associations between pre-infusion iron status and haematological variables

Those patients (Table 2) who were categorised as truly iron deficient (ferritin < 30 μg/L and CRP ≤ 5 mg/L, *n* = 18) had the lowest median pre-infusion haemoglobin (100 mg/L, range 77 to 118). They had the greatest median increase in haemoglobin between infusion and surgery (24 mg/L, range − 6 to 48) and the highest median postoperative day 1 haemoglobin (108 mg/L, range 87 to 133).

Those patients who were categorised as FID (ferritin < 30 μg/L and CRP > 5 mg/L, *n* = 17) had the second lowest median pre-infusion haemoglobin (102 mg/L, range 65 to 122), the second greatest median increase in haemoglobin between infusion and surgery (15 mg/L, range − 15 to 63), and the second highest median postoperative day 1 haemoglobin (103 mg/L, range 87–138).

Those patients who were categorised as having anaemia of inflammation (ferritin ≥ 30 μg/L and CRP > 5 mg/L, *n* = 17) had a higher median pre-infusion haemoglobin than either of the two iron-deficient groups (110 mg/L, range 75 to 117), a lower median increase in haemoglobin between infusion and surgery than either of the two iron-deficient groups (3 mg/L, range − 11 to 34), and a lower median postoperative day 1 haemoglobin than either of the two iron-deficient groups (102 mg/L, range 74–113).

Finally, those patients who were categorised as having anaemia of another cause or without evidence of iron deficiency (ferritin < 30 μg/L and CRP ≤ 5 mg/L, *n* = 18) had a similar median pre-infusion haemoglobin to those with anaemia of inflammation (110 mg/L, range 103 to 115). They had a median decrease in haemoglobin between infusion and surgery (− 5 mg/L, range − 10 to 8) and the lowest median postoperative day 1 haemoglobin (95 mg/L, range 85 to 99). Although this group were not defined as iron deficient by protocol, none were found to be vitamin B12 or folate deficient.

### Associations with post-iron infusion change in haemoglobin

At multivariate linear regression (Table 3), total parenteral iron dose (*p* = 0.034), time from infusion to surgery (*p* < 0.001), haemoglobin prior to iron infusion (*p* = 0.002), and CRP- and ferritin-based iron status group (*p* = 0.006) all remained independently associated with post iron infusion change in haemoglobin.

**Table 3:** Univariate and multivariate linear regression of factors associated with change in haemoglobin (Δ Hb mg/L) following intravenous iron infusion prior to elective colorectal surgery for cancer (*n* = 47)

Characteristic	Univariate regression coefficient (95% CI)	*p*	Multivariate regression coefficient (95% CI)	*p*
Male sex	− 7.7 (− 17.3 to 1.9)	0.112	–	–
Age	4.7 (− 1.6 to 10.9)	0.140	–	–
ASA	2.0 (− 5.6 to 9.7)	0.594	–	–
TNM stage	8.2 (2.2–14.2)	0.008	–	0.780
Tumour site	1.8 (− 4.9 to 8.6)	0.590	–	–
Total iron dose (mg)	0.02 (0.01–0.03)	0.003	0.01 (0.0–0.2)	0.034
Days from infusion to surgery	0.5 (0.3–0.7)	< 0.001	0.4 (0.2–0.6)	< 0.001
Pre-iron infusion Hb (mg/L)	− 0.8 (− 1.1 to − 0.6)	< 0.001	− 0.5 (− 0.7 to − 0.2)	0.002
Pre-iron infusion CRP and ferritin group	− 6.9 (− 11.50 to − 2.19)	0.005	− 4.7 (− 7.9 to − 1.47)	0.006

*AOI* anaemia of inflammation, *ASA* American Society of Anesthesiology, *CRP* C-reactive protein (mg/L), *Fer* ferritin (μg/L), *FID* functional iron deficiency, *Hb* haemoglobin (g/L), *IDA* iron deficiency anaemia, *IV* intravenous, *POD* postoperative day

## Discussion

The results of the present study suggest that, in patients with primary operable colorectal cancer found to be anaemic at diagnosis, preoperative iron infusions improved pre- and postoperative haemoglobin in the truly iron deficient, but not those without evidence of iron deficiency. Those patients with low ferritin in the presence of inflammation, consistent with the presence of functional iron deficiency, had an intermediate haemoglobin response to iron infusion. Those with both high ferritin and high CRP, consistent with the presence of anaemia of inflammation, had a negligible haemoglobin response to iron infusion. In addition, a higher dose of iron, a longer time delay between infusion and surgery, and a lower haemoglobin before infusion were associated with greater increases in haemoglobin after iron infusion.

These findings are in-keeping with the hypothesis that in a proportion of anaemic colorectal cancer patients, the host inflammatory response to cancer leads to either a state of functional iron deficiency anaemia or anaemia of inflammation (Tokunaga et al. 2018). It would appear from this small cohort that functional iron deficiency can, to an extent, be improved by parenteral iron supplementation. It may be that in this group, the dose and parenteral route of iron supplementation overcomes hepcidin-mediated iron sequestration and inhibition of absorption of dietary iron; however, this does not fully explain the lesser response to iron seen in patients with high levels of inflammation (Munoz et al. 2011). Although previous studies have noted success in the correction of preoperative anaemia with oral iron in true iron deficiency (Lidder et al. 2007), it is less clear whether oral supplementation is of use in the FID group.

Conversely, those patients with haematological and biochemical measures consistent with anaemia of inflammation (normal ferritin, raised CRP) appear to have anaemia which is resistant to correction by iron infusion as it is likely caused by other factors. The prevalence of this condition in colorectal cancer must be recognised and clinicians should be cognoscente of this when determining how to optimise their patients in the perioperative period. Preoperative systemic inflammation has been reported to be associated with co-morbidity, postoperative complications, and poorer long-term survival in patients with colorectal cancer, and it may be that preoperative anaemia is an important and related factor (Dolan et al. 2018).

Allogeneic blood transfusions have been associated with increased postoperative infective complications, increased disease recurrence, and poorer survival following surgery for colorectal cancer (Acheson et al. 2012; Pang et al. 2019). Although the mechanism is not fully understood, one key hypothesis is that allogenic blood causes host adaptive immune suppression (Blumberg and Heal 1994), even when using modern leucodepleted packed red cells (Mynster et al. 2000). Therefore, current trends in perioperative medicine and blood management strategies aim to reduce the need for perioperative allogeneic blood transfusion, with preoperative iron supplementation forming a key strategy (Ng et al. 2015). Parenteral iron is not without potential for harm, being associated with a small risk of anaphylaxis. In addition, there is now some evidence that excess iron may be associated with negative oncologic outcomes (Xue et al. 2016; Greten 2016; Radulescu et al. 2012). It is possible that such an effect may be compounded in patients with an existing host systemic inflammatory response, although further studies are required to examine this matter. In attempting to reduce perioperative transfusion, there is evidence from this study, unsurprisingly, that the best haemoglobin response to parenteral iron replacement is in those with true iron deficiency. Further strategies preventing and addressing other causes of anaemia in colorectal cancer are required. This can only be achieved by better understanding of the causes of anaemia in this patient group. It is possible that treatment of inflammation may release checks on erythropoiesis and should be the subject of further study.

It was of interest that tumour site appeared to have an impact on pre-iron infusion iron status defined by CRP and ferritin in this protocol. Almost all patients grouped as having functional iron deficiency and anaemia of inflammation had right colonic lesions, as opposed to the significant proportion of patients with rectal cancer in the true iron-deficient group. The link between right colonic tumours and the presence of both local and systemic inflammation has been documented previously (Patel et al. 2018), as has the association between right colonic tumours and normocytic anaemia (Vayrynen et al. 2018). Therefore, the host interaction with these proximal colonic lesions seems to drive systemic inflammatory driven changes in iron and erythrocytes.

This study has a number of limitations. The number of patients is small, increasing the risk of type 2 error. No data was available regarding patients who had received oral iron supplementation in either primary or secondary care before diagnosis or surgery, increasing the possibility of selection bias and preventing a useful comparison between different routes of iron supplementation in patients with functional iron deficiency or anaemia of inflammation. Furthermore, there were no data examining patients with functional iron deficiency or anaemia of inflammation who did not receive a preoperative iron infusion. In addition, a number of patients who were anaemic but without evidence of inflammation or iron deficiency received an iron infusion, against the unit protocol. Again, this could suggest selection bias, but in terms of an observational study, provided an additional interesting group of patients for comparison in a real-world study, with findings confirming they fail to respond to iron infusion as hypothesised. Although data did exist relating to perioperative blood transfusion in this cohort, the lack of an established transfusion protocol meant that this was not included in the analysis. Finally, there was no reliable recording of estimated operative blood loss in the cohort, which may lead to bias with regard to the analysis of postoperative haemoglobin.

In summary, this observational study reports a reduced efficacy of preoperative parenteral iron supplementation in patients thought to have anaemia of inflammation, and to a lesser extent functional iron deficiency, in patients undergoing surgery for colorectal cancer, compared with those who were truly iron deficient. This study illustrates that iron infusion is of greatest benefit to the truly iron-deficient patient in the absence of preoperative inflammation. Given that the presence of anaemia and inflammation are in fact much more common in the preoperative colorectal cancer population than iron deficiency anaemia alone, these results suggest that this area requires further focused study. It may be that alternative preoperative haemoglobin optimisation methods, for example targeting the underlying host inflammatory response to cancer, may prove to be of more use in these groups of patients than targeting all patients with low ferritin levels with preoperative iron infusions.

## Data Availability

The datasets generated and/or analysed during the current study are not publicly available due to the small number of patients included and therefore the possibility that a patient may be identified even from otherwise anonymised data. Limited data are available from the corresponding author on reasonable request**.**

## Abbreviations

AJCC: American Joint Committee on Cancer
ASA: American Society of Anesthesiology
AOI: Anaemia of inflammation
CI: Confidence interval
CNS: Cancer nurse specialist
CRC: Colorectal cancer
CRP: C-reactive protein
EPO: Erythropoietin
EQA: External quality assurance
ERAS: Enhanced recovery after surgery
F: Female
FBC: Full blood count
FID: Functional iron deficiency
GP: General practitioner
Hb: Haemoglobin
IDA: Iron deficiency anaemia
IQC: Internal quality control
IV: Intravenous
M: Male
MCV: Mean corpuscular volume
mGPS: Modified Glasgow Prognostic Score
POD: postoperative day
SDAU: Same day admission unit
TNM: Tumour nodes metastasis staging
TSAT: Transferrin saturation
WHO: World Health Organization
